# Immunosenescence and gender: a study in healthy Cubans

**DOI:** 10.1186/1742-4933-10-16

**Published:** 2013-04-30

**Authors:** Beatriz García Verdecia, Danay Saavedra Hernández, Patricia Lorenzo-Luaces, Teresita de Jesús Badía Alvarez, Idrissa Leonard Rupalé, Zaima Mazorra Herrera, Tania Crombet Ramos, Agustín Lage Dávila

**Affiliations:** 1 Department of Clinical Immunology, Center of Molecular Immunology, P.O. Box 16040, 216 St. Havana, Cuba

## Abstract

**Background:**

The progressive decline in the immune function during ageing is termed immunosenescence. Previous studies have reported differences between males and females in the distribution and cell responses of lymphocyte subsets. Most studies of immunosenescence have been done in populations of industrialized countries living in a rather cold environment, and facing lower antigenic challenges such as Cytomegalovirus (CMV). The aim of this study was to determine the effect of ageing on lymphocytes in a population with a high prevalence of CMV infection in all ages, and to compare gender differences related to the immunosenescence markers.

**Results:**

Different populations of peripheral blood leukocytes from healthy young and old IgG-CMV seropositive individuals were examined using flow cytometry. With age, the number and frequency of B cells and T cells significantly decreased, while highly differentiated T cells increased. Such changes were different in males and females. The age-associated decline of less differentiated lymphocyte subsets (CD19, CD4 and CD8 cells) and the increase of highly differentiated T cells were more prominent in females. In males, there were no significant changes in CD19, CD4 and CD8 subsets but there was a significant increase in the proportion of highly differentiated T cells.

**Conclusion:**

Shifts in lymphocyte subsets distribution were influenced by age and gender in an IgG-CMV seropositive population. These results suggest different patterns of immunosenescence in respect to gender differences. These patterns could have implications in the design of immunotherapy in the elderly.

## Introduction

The function of the immune system declines with age leading to increased susceptibility to infectious diseases, cancer and poor response to vaccination [1,2]. This decline is termed “Immunosenescence” [3].

Immunosenescence is related to thymus involution, reduction in the generation of naïve T-cell populations, changes in the peripheral T lymphocyte compartments and reduced functional immune competence. The nature of these changes is associated with exposure to environmental challenges, such as stress and pathogens [4].

Age-associated changes in lymphocyte subpopulations have been documented in diverse contexts. The Swedish octogenarian study (OCTO-immune), showed an increasing number of individuals with CD4:CD8 ratio less than 1, driven by the decrease in CD4 T cell subsets and/or expansion of CD8 T cells. The increase in CD8 and its relationship with cytomegalovirus (CMV) infection suggest that the infection with this virus might be a contributor to this significant expansion of CD8 T cells [5].

In Britain, Akbar and coworkers identified the progressive stages in T cell differentiation by sequential changes in the expression of surface markers, such as CD45RA, CD28, CD27, and CCR7 [6]. The proportion of most differentiated T cells in both CD4+ and CD8+ populations, were designated as CD28-, CD27-, CCR7- by another group in France [7].

Age-related changes in CD4 T cells have been less documented than in CD8 cells. However, there is also evidence of an increase of CD45RA+CD27-CD4+ T cells in older subjects [8], especially in CMV-infected subjects. These cells are mostly CMV-specific, but there is also an increase in effector CD4+ T cells that are specific for other viruses, i.e. EBV, HSV, and VZV [8].

One of the fundamental manifestations of immunosenescence is a steady decrease of functional thymus tissue which begins after birth, accelerates with puberty and is almost completed by the age of 50 [9,10]. A dramatic decline in peripheral naïve T cells in both, CD4+ and CD8+ subsets, have been observed in elder people [11]. The kinetics of thymus involution could be related to the genetic background of human populations and is also influenced by sex hormones, such as estrogen and testosterone. The thymus made fewer new cells with age in both sexes, but women still had higher levels of new T cells than men of the same age [12]. Epidemiological differences between sexes (such as weight, lifestyle, smoking, and medication) could also account for differences observed [13].

Recent studies in healthy humans considered the differences in immunosenescence between males and females [14,15].

Additionally, immunosenescence is at least partially driven by the chronic antigenic load, which is obviously different in diverse environments. These facts support the hypothesis that the kinetics of immunosenescence could be different in each country and therefore should be characterized in every specific context as part of the information needed for a scientifically based public health policy, especially in countries with aged populations.

Most studies attempting to assess immunosenescence have focused on a single population, mostly “WEIRD” (ie Western, educated, industrialized, rich, democratic). These are not necessarily representative of the majority of the world’s population [16]. There is a scarcity of data about immunosenescence in developing countries and tropical climate.

This article presents findings from a Cuban population study aimed to investigate the effect of age and gender on lymphocyte populations in healthy humans. Moreover, Cuba could become a kind of natural experiment providing one of the rare situations in which long life expectancy, low infant mortality and intensive vaccination coverage, typical of northern wealthy populations, are coincident with the high antigenic load of a tropical environment.

## Results

### Prevalence of CMV-IgG seropositivity

CMV-IgG seropositivity was determined in all individuals (n=112). The overall prevalence of seropositive individuals was 90.1% ( n=101). Antibodies against CMV were found in more than 90% of old individuals no matter the gender. However in younger ages, there was a higher frequency of seropositive in males (93.3%) than in females (73.6%), although statistical significance was not achieved (Fisher test, *p*>0.05).

Eleven subjects (8 females and 3 males) distributed within both age groups, were CMV-IgG seronegatives. As a consequence of their low prevalence in each age/gender group, it was not possible to establish comparisons between seronegative and seropositive individuals in each group. In consequence, they were omitted in the subsequent analysis.

### Significant decline of total lymphocyte counts with age in CMV seropositive Cubans is driven by the female group

**Figure 1:**
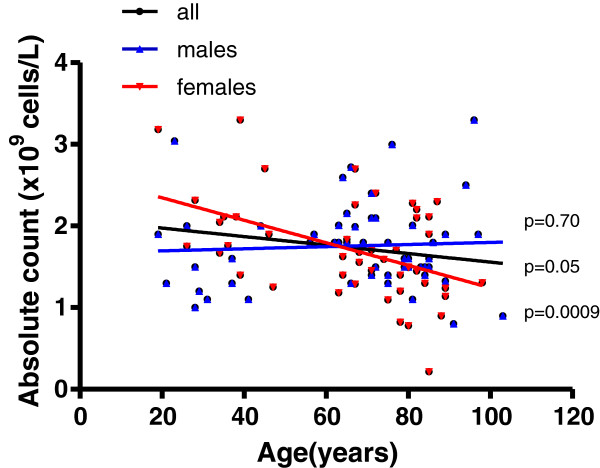
**Distribution of lymphocytes counts from CMV-IgG seropositive individuals.** The linear regression results for males (blue line, n=53) and female (red line, n=47), the corresponding p values are shown on the graphs nearby each regression line, *p*<0.05 indicates statistical differences.

There was a significant decline with age of the total lymphocyte counts in CMV seropositive individuals for the whole population. However, this decline was significant only for females (Figure 1), showing higher lymphocyte counts in younger ages than the young males (Man Whitney test, p<0.05). In contrast, males had the lymphocyte counts practically constant at all ages.

**Figure 2:**
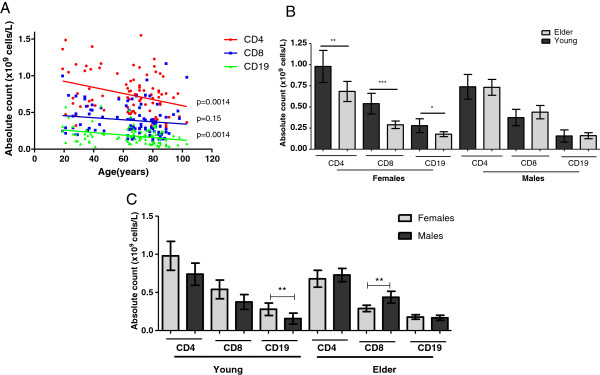
**Absolute counts of B and T cells subsets in healthy Cuban subjects. A**) CD4, CD8 and CD19 lymphocytes distribution by ages. The cell markers that PBL were stained for are represented on the Y axis in different colors. The linear regression results for CD19+ (green line, n=94, CD8+ (blue line, n=99) and CD4+ cells (red line, n=94) and the *p* values are shown on the graphs nearby each regression line, *p*<0.05 indicates statistical differences. **B**) Absolute counts of CD4, CD8 and CD19 lymphocytes subsets by gender and group of age, significant differences between young (dark gray) and aged individuals (light gray) in each case are labeled. **C**) Differences between males (dark gray) and females (light gray) by age groups. (*) indicate significant differences by Mann Whitney test (*p*<0.05).

When lymphocytes were further subdivided into B cells (CD19+) and T cells (CD4+ and CD8+), the decline in lymphocyte counts with age was evident only in CD4+ and CD19+ subgroups (Figure 2A). Significant decline in counts of each subset with age were observed only in females (Figure 2B). In fact, females displayed higher counts of T and B cells than males in young ages, being significant for B cells (Mann Whitney test *p*<0.05). Conversely, in old ages males had higher quantities of CD8 T cells than females (Figura 2C). The counterbalance between genders justifies the no variation in CD8T cell counts with age.

No changes in the ratio of CD4+:CD8+ T cells with age were observed in the CMV seropositive individuals included in the previous analysis. None of the seronegative individuals showed inversion in the CD4+:CD8+ ratio. Within seropositive ones there was a low frequency of inversion (8.9%, n=9). A higher prevalence of an inverted CD4+:CD8+ ratio was found in the aged group (10.9% vs 3.6% in the youngest) and the highest frequency was observed in elder males. However, no significant differences were found applying a Fisher test.

### Significant increase in the proportion of activated and differentiated CD8 T cells with age

**Figure 3:**
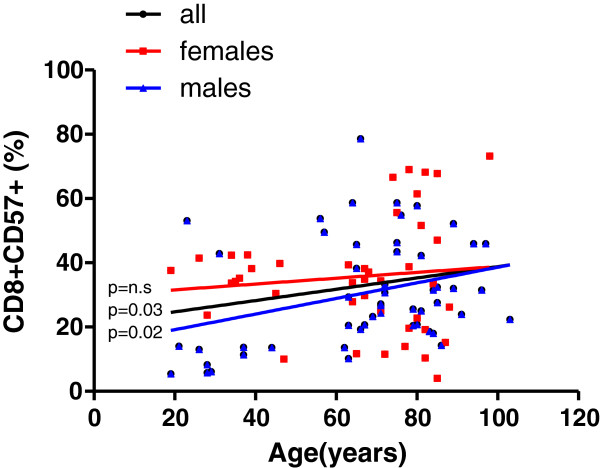
**Frequency distribution of CD57+ cells within total CD8+ T cells.** The cell markers that PBL were stained for are shown on the Y axis. The linear regression results are represented in different colors for all individuals (black line, n=94), males (blue line, n=52) and females (red line, n= 42). The *p* values for all individuals are shown on the graphs nearby the regression line, *p*<0.05 indicates statistical differences.

The proportion of activated CD8+ T cells increased with age, but this increase was significant only in males (Figure 3).

**Figure 4:**
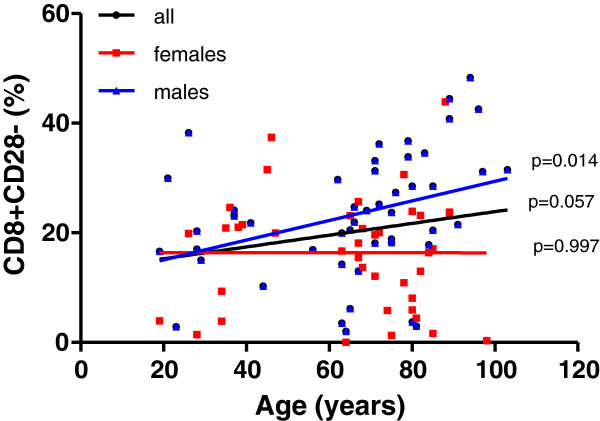
**Frequency distribution of CD28- cells within total CD8+ T cells.** The cell markers that PBL were stained for are shown on the Y axis. The linear regression results are represented in different colors for all individuals (black line, n=88), males (blue line, n=48) and females (red line, n= 40). The *p* values for all individuals are shown on the graphs nearby the regression line, *p*<0.05 indicates statistical differences..

Moreover, the proportion of differentiated CD8+ T lymphocytes increased with age (Figure 4). In addition to activated CD8+T cells, the increase was only significant in males. In contrast, women kept the proportions of CD8+ CD57+ and CD8+CD28- T cells nearly constant at all ages.

### Variation of terminally differentiated CD45RA+ CD28- T cells with age and gender

**Figure 5:**
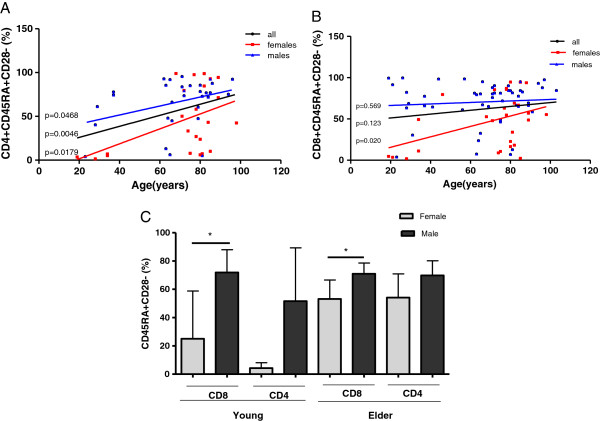
**Frequency of highly differentiated T cells according to ages and genders. A**) Linear regression shows the percentages of CD45RA+/CD28- population within total CD4+ T cells for all individuals (black line, n=54), males (blue line, n=31) and females (red line, n=23), *p* values are shown on the graphs to each regression line. **B**) Linear regression shows the percentages of the CD45RA+/CD28- population within total CD8+ T cells for all individuals (black line, n= 79), males (blue line, n=51) and females (red line, n=28), *p* values are shown on the graphs to each regression line. p<0.05 indicates statistical differences. **C**) Frequency of highly differentiated T cells (within CD4+ and CD8+ subsets) in males and females by age groups. Differences between males (dark gray) and females (light gray) are represented. * indicates significant differences by Mann Whitney test (*p*<0.05).

The expression of CD45 together with the loss of CD28 is a marker of terminally differentiated T lymphocytes [17]. The proportion of this population in CD4+T cells increased with age both in males and females (Figure 5A). For CD8+ T cells this increase in terminally differentiated cells with age was significant only in females (Figure 5B).

Cuban males showed a higher proportion of terminally differentiated T cells than females since youth ages. In fact, young males displayed higher frequencies of CD45RA+CD28- T cells than young females within both CD4+ (*p*=0.063) and CD8+ (*p*=0.015) T cell subsets (Mann Whitney test *p*<0.05). However in elder populations, significant differences (*p*=0.027) were found only within CD8+ T lymphocytes. Indeed, elder males had a higher frequency of CD8+CD45RA+CD28- than elder females (Figure 5C).

## Discussion

Changes in cells of the immune system related to age have been the subject of many studies in the last two decades [18-22].Most published studies document a decrease in the number and proportion of naïve CD8 T cells in aged individuals together with a reciprocal increase in memory CD8 T cells [4,23]. These facts have been frequently associated to a seropositive response to CMV [24]. There are also recent data showing a decline in peripheral naive CD4 T cells number with age [11] and a recent study showed that CMV infection significantly increases the proportion of effector memory-like CD4+ T cells in elder humans [8].

Another study of 1,468 community-dwelling elderly Latinos, showed that increasing CMV IgG antibody titers were associated with the overall cause of mortality even after adjusting for various covariates such as age, gender, education, and baseline health conditions [25]. The CMV persistent infection has been considered as one of the major drivers of immunosenescence [26] and such study also contributes to the growing evidence that CMV immune response is related to the deleterious processes involved in aging.

The hallmarks of immunosenescence would be affected by the history of the individual’s exposure to pathogens [16]. The Cuban population could be a particular case worth to study, where exceptionally the high antigenic load typical of a developing country in the tropical belt is coincident with low infant mortality, high life expectancy [27] and an aged demographic pyramid [28], as a consequence of social interventions.

The high prevalence of CMV infection in tropical countries has been attributed to several sociocultural behaviors that may predispose to increased transmission of human CMV in early ages of life [29]. A high prevalence of seropositivity to cytomegalovirus in Cubans, after hemodialysis, HIV infection and renal transplants has been previously described [30-32]. The present study is the first one to evaluate the immunosenescence markers in Cubans. We also confirmed a high seroprevalence of CMV infection in our population since young ages, but in this case in healthy individuals.

We found age-associated decreases in the counts of total lymphocytes, both in B (CD19+) and T cells (CD4+ and CD8+ T cells). These findings are very similar to previous results showing a significant decline of total lymphocytes with age [33] as a consequence of the contraction of T [34] and B cell populations [35]*.* In addition, we found gender differences consisting of a significant decline in the lymphocytes number with age, in females, and not in males.

Differences in the immune system functioning in males and females have been previously described [36,37]. Such differences may occur due to the opposing ways in which hormones act [38,39]. In fact, Th1 and Th2 responses appear affected by androgenic and estrogenic preponderance, respectively. While androgens favor the development of a Th1 response and the activation of CD8 cells [40]; estrogens seem to direct the immune system towards a Th2 dominance, where B lymphocytes are activated [39]. Those findings are in line with the higher levels of B cells found in Cuban females, and in the increased frequency of differentiated CD8 T cells in males since young ages.

Furthermore, hormonal differences seem the most probable cause of gender-related changes in the rate of thymus involution. Male sexual hormones have been shown to accelerate thymus involution more profoundly than female hormones, according to reports of improved thymus function in male gonadectomized mice [41]. While the thymus made fewer new cells with age in both sexes, women still had higher levels of new T cells than men of the same age [12]. Epidemiological differences between the sexes (such as weight, lifestyle, smoking, and medication) could also account for the differences observed [13]. In addition, several studies in mice showed a more vigorous humoral and cellular immune responses in females than in males [42,43], and a superior ability of females to fight infections [44,45]. All these findings are in line with our results on gender differences in B and T cell compartments.

In our study the main markers of immunosenescence in CMV seropositive males were those related to the proportion of CD8 differentiated T cells. The age-associated increase in the proportion of CD8+/CD57+ cells, expressing down modulation of cytotoxic activity, and in terminally differentiated CD8+/CD28- T cells were only observed in males. Lenkei and Andersson reported a correlation of CMV antibody titers with lymphocyte activation status and CD57+ expression [46]. Some studies from Merino et al. reported that expansion of CD8+CD57+CD28- cells was related to age, and also to CMV carrier status possibly related to repeated antigenic stimulation [47]. However these previous studies did not report influence of gender on differentiation markers.

Additionally, It is known that during ageing and after CMV infection there is an increase of highly differentiated CD45RA re-expressing T cells within both the CD4 and CD8 compartments [48-50], This observations may reflect the impact of thymic involution compounded with persistent CMV infection during ageing [2]. We also found increasing proportions of terminally differentiated CD8+/CD45RA+/CD28- T cells with age, but only significant in females, while males showed a higher proportion of these cells compared to females of young ages. Concerning terminally differentiated CD4 T lymphocytes, the results showed an age-associated increase in both sexes and a higher proportion of CD4+/CD45RA+/CD28- T cells also in males.

There have been many studies on the CMV-specific CD8+ T cell population [24,51-53] and some about the characteristics of CMV-specific CD4+ T cells describing the impact that CMV infection has in shaping the T cell pool [11,54,55]. The majority of highly differentiated T cell populations in CMV-infected subjects are CMV specific but there are also increased numbers of these effector T cells that are specific for other viruses, i.e. EBV, HSV and VZV [8]. This put forward that CMV infection may drive a global increase in T cell differentiation suggesting a bystander phenomenon [8]. Alternatively, this could be due to an epidemiological coincidence of infections with diverse viruses. This hypothesis deserves further testing in Cuban population where a high seroprevalence of herpesviruses (CMV, herpes simplex type 1 virus (HSV) and Epstein Barr virus(EBV) infection has been previously described [30-32,56]*.*

To our knowledge, the impact of gender in shaping the T cell pool in CMV infected healthy humans has not been described before. The lower proportion of CD45RA+ CD28- T cells (both CD4 and CD8) exhibited by females during young ages and the high frequency of highly differentiated CD8 T cells sustained since young ages in males, is probably linked to gender differences in the contribution of thymic emigrants to the peripheral T cell pool with age previously reported by different authors [57-59]. This is also shown by persistent higher values of signal-joint T cell receptor rearrangement excision circles (sjTREC) in females between the ages of 20 and 50, and suggesting that the thymic output is prolonged at higher levels in females as compared with males [12]. On the other hand, a very recent and unpublished study in Cuban patients (including more than 50% of pediatric samples) with mononucleosis syndrome, found higher seroprevalence of CMV (>79%) and EBV (64,8%) than of HSV (author personal communication). Consequently with this finding, we think that a higher exposure to antigenic loads +since infancy in Cuban population could drive a premature immunosenescence. This phenomenon could occur essentially in males, which could be potentially less protected, since male sexual hormones have been shown to accelerate thymus involution more deeply than female hormones [41].

The gender contrast in our study, points to a conservation of the output of naive cells until advanced ages in females, and a higher impact of antigen-driven differentiation of T cells in males probably due to genetic and environmental factors related to the control of lifespan. In fact, recent findings indicate that gender is a major variable in the genetics of longevity, suggesting in turn that men and women follow different strategies to reach longevity [60,61]. These gender differences in the immunosenescence process remain significant even inside a population with high prevalence of CMV seropositivity in both sexes. Future studies should include the analysis of naive T cells and TRECS in both gender groups.

In elder population the differences in gender were found mainly for the differentiated CD8+T cells, where males showed again the highest proportion. However, in general we found a broader spectrum of changes with age in females rather than in males. Conversely, other studies in humans showed that changes observed with ageing were more apparent in males [14,15] but the evidences are statistically significant only for a decrease of CD8+ alpha beta T cells and for an increase of the effector memory cells (CD3+CD45RA-CCR7-) [15]. Wikby et al examined immune parameters for the adult lifespan and some of their major findings were a higher frequency of individuals with an inverted CD4/CD8 ratio and lower levels of CD3+CD4+ and CD8+CD45RA+CCR7+ cells in men than in women [14]. It should be noticed that these studies evaluated different markers, and targeted human populations with lower life-long antigenic load, which could explain such dissimilar results.

Gender differences in the performance of the immune system have been suggested long time ago by epidemiological evidences such as the higher incidence of autoimmune diseases in females, and atherosclerosis-related diseases (linked to chronic inflammation) in males. However, the interplay among hormones, immunity and inflammation is so complex that resists any simple mechanistic interpretation.

Cancer vaccine clinical trials from our own group have previously shown that age is related to the survival advantage conferred by therapeutic vaccination with an immunogenic preparation containing human Epidermal Growth Factor [62].

Our data, together with previous findings on the role of gender in the genetic determinants of longevity [61,63] and the gender-specific age-related remodeling of the cytokine network [64] indicate the need for deeper studies on gender-specific immunosenescence phenotypes to further explore their impact on immunotherapy and the potential relevance for personalized medicine. Such studies in naive and treated cancer patients are currently ongoing.

## Methods

### Subjects

Peripheral blood samples were collected from healthy volunteers by venipuncture.

Subjects were regarded as being healthy if they had no acute illness, and were on no medication other than anti-hypertensive medication, and had no serious prior illnesses. The age and sex distribution of the subjects are summarized in Table 1.

**Table 1:** Age and gender distribution of healthy volunteers

**Age group**	**Total**	**Male**	**Female**
Young (19–57)	34	15	19
Old (61–103)	78	41	37
Total	112	56	56

Individuals younger than 60 years old were defined as young (median age 34 years old); while individuals older than 60 years old were defined as elder (median age 77.5 years old).

### White blood cells collection and staining for flow cytometry

Red cells were isolated from whole blood with lysing solution (NH_4_Cl, EDTA (tetrasodium), KHCO_3_). White blood cells (WBCs) were washed twice with Citometry solution (PBS, BSA, Azide 20%). Specific antibodies against CD3, CD4, CD8, CD19, CD45RA, CD28 and CD57 molecules, conjugated either to FITC, PE and Cy5, were purchased from Serotec and used for staining.

For surface staining, WBCs (1 × 10^5^ cells in 100 μL Citometry solution) were incubated with 1 μL of the antibody in the dark at 4°C for 20 min. Subsequently, cells were washed twice and analyzed on a three-color flow cytometer (FACS Scan), with gating on the total lymphoid populations. Samples were obtained and studied individually. All the data generated was obtained from fresh samples.

### CMV serology

Serum IgG antibodies to CMV were measured with an enzyme immunoassay (ETI-CYTOK-G PLUS P002033, DiaSorin) and expressed as arbitrary units/ml (UI/mL). A tested result higher than 0.4 UI/mL was considered positive for the presence of CMV-specific IgG antibodies, as per the manufacturer’s guidelines.

### Statistical analysis

The relationship between age in years and the absolute counts of cells with different cell surface markers was studied by linear regression analysis. Statistical significance between groups was evaluated using nonparametric Kruskal-Wallis test within One-way ANOVA or Mann Whitney test. Post hoc paired comparisons with Dunn’s Multiple Comparison Test were done for the variables that showed differences between groups after ANOVA analysis. All statistical analyses were performed with SPSS v15.0 and GraphPad 5. The statistical data were considered significant if *p* < 0.05.

## Abbreviations

CMV: Cytomegalovirus; WBCs: White blood cells; PBL: Peripheral blood Lymphocytes.

## Competing interest

The authors declare that they have no competing interests.

## Authors’ contributions

TB performed the analysis, BG and DS supervised the FACS analysis, BG and IL was responsible for recruiting, consenting subjects and contributed to the recruitment of subjects and to the development of the study, PL performed and supervises the statistical analysis, AL, ZM and TC has overall responsibility for the project, BG, DS and AL has overall responsibility for writing the paper. All authors read and approved the final manuscript.

## References

[B1] Shin H Wherry EJ CD8 T cell dysfunction during chronic viral infection Curr Opin Immunol 2007 19 4 408 415 10.1016/j.coi.2007.06.004 17656078

[B2] McElhaney JE Effros RB Immunosenescence: what does it mean to health outcomes in older adults? Curr Opin Immunol 2009 21 4 418 424 10.1016/j.coi.2009.05.023 19570667 PMC2725188

[B3] Pawelec G Solana R Immunosenescence Immunol Today 1997 18 11 514 516 10.1016/S0167-5699(97)01145-6 9386344

[B4] Wikby A Johansson B Olsson J Lofgren S Nilsson BO Ferguson F Expansions of peripheral blood CD8 T-lymphocyte subpopulations and an association with cytomegalovirus seropositivity in the elderly: the Swedish NONA immune study Exp Gerontol 2002 37 2–3 445 453 11772532 10.1016/s0531-5565(01)00212-1

[B5] Olsson J Wikby A Johansson B Lofgren S Nilsson BO Ferguson FG Age-related change in peripheral blood T-lymphocyte subpopulations and cytomegalovirus infection in the very old: the Swedish longitudinal OCTO immune study Mech Ageing Dev 2000 121 1–3 187 201 11164473 10.1016/s0047-6374(00)00210-4

[B6] Akbar AN Fletcher JM Memory T cell homeostasis and senescence during aging Curr Opin Immunol 2005 17 5 480 485 10.1016/j.coi.2005.07.019 16098721

[B7] Appay V van Lier RA Sallusto F Roederer M Phenotype and function of human T lymphocyte subsets: consensus and issues Cytometry A 2008 73 11 975 983 18785267 10.1002/cyto.a.20643

[B8] Libri V Azevedo RI Jackson SE Di Mitri D Lachmann R Fuhrmann S Vukmanovic-Stejic M Yong K Battistini L Kern F Cytomegalovirus infection induces the accumulation of short-lived, multifunctional CD4+CD45RA+CD27+ T cells: the potential involvement of interleukin-7 in this process Immunology 2011 132 3 326 339 10.1111/j.1365-2567.2010.03386.x 21214539 PMC3044899

[B9] Steinmann GG Klaus B Muller-Hermelink HK The involution of the ageing human thymic epithelium is independent of puberty. A morphometric study Scand J Immunol 1985 22 5 563 575 10.1111/j.1365-3083.1985.tb01916.x 4081647

[B10] Aspinall R Andrew D Thymic involution in aging J Clin Immunol 2000 20 4 250 256 10.1023/A:1006611518223 10939712

[B11] Weinberger B Lazuardi L Weiskirchner I Keller M Neuner C Fischer KH Neuman B Wurzner R Grubeck-Loebenstein B Healthy aging and latent infection with CMV lead to distinct changes in CD8+ and CD4+ T-cell subsets in the elderly Hum Immunol 2007 68 2 86 90 10.1016/j.humimm.2006.10.019 17321897

[B12] Pido-Lopez J Imami N Aspinall R Both age and gender affect thymic output: more recent thymic migrants in females than males as they age Clin Exp Immunol 2001 125 3 409 413 10.1046/j.1365-2249.2001.01640.x 11531948 PMC1906152

[B13] Oraei M Aghamohammadi A Rezaei N Bidad K Gheflati Z Amirkhani A Abolhassani H Massoud A Naive CD4+ T cells and recent thymic emigrants in common variable immunodeficiency J Investig Allergol Clin Immunol 2012 22 3 160 167 22697005

[B14] Wikby A Mansson IA Johansson B Strindhall J Nilsson SE The immune risk profile is associated with age and gender: findings from three Swedish population studies of individuals 20-100 years of age Biogerontology 2008 9 5 299 308 10.1007/s10522-008-9138-6 18369735

[B15] Yan J Greer JM Hull R O’Sullivan JD Henderson RD Read SJ McCombe PA The effect of ageing on human lymphocyte subsets: comparison of males and females Immun Ageing 2010 7 4 10.1186/1742-4933-7-4 20233447 PMC2858100

[B16] Pawelec G Hallmarks of human “immunosenescence”: adaptation or dysregulation? Immun Ageing 2012 9 1 15 10.1186/1742-4933-9-15 22830639 PMC3416738

[B17] Akbar AN Henson SM Are senescence and exhaustion intertwined or unrelated processes that compromise immunity? Nat Rev Immunol 2011 11 4 289 295 10.1038/nri2959 21436838

[B18] Effros RB Boucher N Porter V Zhu X Spaulding C Walford RL Kronenberg M Cohen D Schachter F Decline in CD28+ T cells in centenarians and in long-term T cell cultures: a possible cause for both in vivo and in vitro immunosenescence Exp Gerontol 1994 29 6 601 609 10.1016/0531-5565(94)90073-6 9435913

[B19] Huppert FA Solomou W O’Connor S Morgan K Sussams P Brayne C Aging and lymphocyte subpopulations: whole-blood analysis of immune markers in a large population sample of healthy elderly individuals Exp Gerontol 1998 33 6 593 600 10.1016/S0531-5565(98)00033-3 9789736

[B20] Weng NP Aging of the immune system: how much can the adaptive immune system adapt? Immunity 2006 24 5 495 499 10.1016/j.immuni.2006.05.001 16713964 PMC2266981

[B21] Czesnikiewicz-Guzik M Lee WW Cui D Hiruma Y Lamar DL Yang ZZ Ouslander JG Weyand CM Goronzy JJ T cell subset-specific susceptibility to aging Clin Immunol 2008 127 1 107 118 10.1016/j.clim.2007.12.002 18222733 PMC2435295

[B22] Kumar R Burns EA Age-related decline in immunity: implications for vaccine responsiveness Expert Rev Vaccines 2008 7 4 467 479 10.1586/14760584.7.4.467 18444893

[B23] Kern F Khatamzas E Surel I Frommel C Reinke P Waldrop SL Picker LJ Volk HD Distribution of human CMV-specific memory T cells among the CD8pos. subsets defined by CD57, CD27, and CD45 isoforms Eur J Immunol 1999 29 9 2908 2915 10.1002/(SICI)1521-4141(199909)29:09<2908::AID-IMMU2908>3.0.CO;2-8 10508265

[B24] Chidrawar S Khan N Wei W McLarnon A Smith N Nayak L Moss P Cytomegalovirus-seropositivity has a profound influence on the magnitude of major lymphoid subsets within healthy individuals Clin Exp Immunol 2009 155 3 423 432 10.1111/j.1365-2249.2008.03785.x 19220832 PMC2669518

[B25] Roberts ET Haan MN Dowd JB Aiello AE Cytomegalovirus antibody levels, inflammation, and mortality among elderly Latinos over 9 years of follow-up Am J Epidemiol 2010 172 4 363 371 10.1093/aje/kwq177 20660122 PMC2950794

[B26] Solana R Tarazona R Aiello AE Akbar AN Appay V Beswick M Bosch JA Campos C Cantisan S Cicin-Sain L CMV and Immunosenescence: from basics to clinics Immun Ageing 2012 9 1 23 10.1186/1742-4933-9-23 23114110 PMC3585851

[B27] De Vos P Garcia-Farinas A Alvarez-Perez A Rodriguez-Salva A Bonet-Gorbea M Van der Stuyft P Public health services, an essential determinant of health during crisis. Lessons from Cuba, 1989-2000 Trop Med Int Health 2012 17 4 469 479 10.1111/j.1365-3156.2011.02941.x 22296108

[B28] Coyula M Havana: aging in an aging city MEDICC Rev 2010 12 4 27 29 21048541 10.37757/MR2010.V12.N4.6

[B29] Kouri V Correa CB Verdasquera D Martinez PA Alvarez A Aleman Y Perez L Golpe MA Someilan T Chong Y Diagnosis and screening for cytomegalovirus infection in pregnant women in Cuba as prognostic markers of congenital infection in newborns: 2007-2008 Pediatr Infect Dis J 2010 29 12 1105 1110 10.1097/INF.0b013e3181eb7388 20622711

[B30] Marrero M Alvarez M Suarez L Diaz-Jidi M Kouri V Serologic response to some herpesviruses in a group of HIV infected patients Rev Cubana Med Trop 1992 44 3 208 211 9768218

[B31] Resik S Enamorado A Tallo Y Suarez C Kouri V Acosta B Garcia S Prevalence of antibodies against herpes simplex virus, Epstein-Barr virus and cytomegalovirus in a group of patients after hemodialysis Rev Cubana Med Trop 1999 51 3 172 176 10887583

[B32] Glenn J Cytomegalovirus infections following renal transplantation Rev Infect Dis 1981 3 6 1151 1178 10.1093/clinids/3.6.1151 6281874

[B33] Dorshkind K Swain S Age-associated declines in immune system development and function: causes, consequences, and reversal Curr Opin Immunol 2009 21 4 404 407 10.1016/j.coi.2009.07.001 19632102 PMC2742656

[B34] Bender BS Tallman E The heterogeneity of the age-related decline in immune response: impairment in delayed-type hypersensitivity and cytotoxic T-lymphocyte activity occur independently Exp Gerontol 1992 27 3 347 354 10.1016/0531-5565(92)90061-4 1639154

[B35] Cancro MP Hao Y Scholz JL Riley RL Frasca D Dunn-Walters DK Blomberg BB B cells and aging: molecules and mechanisms Trends Immunol 2009 30 7 313 318 10.1016/j.it.2009.04.005 19540810 PMC2766868

[B36] Grossman CJ Regulation of the immune system by sex steroids Endocr Rev 1984 5 3 435 455 10.1210/edrv-5-3-435 6381037

[B37] Pietschmann P Gollob E Brosch S Hahn P Kudlacek S Willheim M Woloszczuk W Peterlik M Tragl KH The effect of age and gender on cytokine production by human peripheral blood mononuclear cells and markers of bone metabolism Exp Gerontol 2003 38 10 1119 1127 10.1016/S0531-5565(03)00189-X 14580865

[B38] Whitacre CC Sex differences in autoimmune disease Nat Immunol 2001 2 9 777 780 10.1038/ni0901-777 11526384

[B39] McCarthy M The “gender gap” in autoimmune disease Lancet 2000 356 9235 1088 10.1016/S0140-6736(05)74535-9 11009154

[B40] Beagley KW Gockel CM Regulation of innate and adaptive immunity by the female sex hormones oestradiol and progesterone FEMS Immunol Med Microbiol 2003 38 1 13 22 10.1016/S0928-8244(03)00202-5 12900050

[B41] Hince M Sakkal S Vlahos K Dudakov J Boyd R Chidgey A The role of sex steroids and gonadectomy in the control of thymic involution Cell Immunol 2008 252 1–2 122 138 18294626 10.1016/j.cellimm.2007.10.007

[B42] Ansar Ahmed S Penhale WJ Talal N Sex hormones, immune responses, and autoimmune diseases. Mechanisms of sex hormone action Am J Pathol 1985 121 3 531 551 3907369 PMC1887926

[B43] Weinstein Y Ran S Segal S Sex-associated differences in the regulation of immune responses controlled by the MHC of the mouse J Immunol 1984 132 2 656 661 6228595

[B44] Lotter H Jacobs T Gaworski I Tannich E Sexual dimorphism in the control of amebic liver abscess in a mouse model of disease Infect Immun 2006 74 1 118 124 10.1128/IAI.74.1.118-124.2006 16368964 PMC1346632

[B45] Mock BA Nacy CA Hormonal modulation of sex differences in resistance to Leishmania major systemic infections Infect Immun 1988 56 12 3316 3319 3182082 10.1128/iai.56.12.3316-3319.1988 PMC259743

[B46] Lenkei R Andersson B High correlations of anti-CMV titers with lymphocyte activation status and CD57 antibody-binding capacity as estimated with three-color, quantitative flow cytometry in blood donors Clin Immunol Immunopathol 1995 77 2 131 138 10.1006/clin.1995.1136 7586720

[B47] Merino J Martinez-Gonzalez MA Rubio M Inoges S Sanchez-Ibarrola A Subira ML Progressive decrease of CD8high+ CD28+ CD57- cells with ageing Clin Exp Immunol 1998 112 1 48 51 10.1046/j.1365-2249.1998.00551.x 9566789 PMC1904936

[B48] Khan N Shariff N Cobbold M Bruton R Ainsworth JA Sinclair AJ Nayak L Moss PA Cytomegalovirus seropositivity drives the CD8 T cell repertoire toward greater clonality in healthy elderly individuals J Immunol 2002 169 4 1984 1992 12165524 10.4049/jimmunol.169.4.1984

[B49] Wills MR Okecha G Weekes MP Gandhi MK Sissons PJ Carmichael AJ Identification of naive or antigen-experienced human CD8(+) T cells by expression of costimulation and chemokine receptors: analysis of the human cytomegalovirus-specific CD8(+) T cell response J Immunol 2002 168 11 5455 5464 12023339 10.4049/jimmunol.168.11.5455

[B50] Derhovanessian E Maier AB Hahnel K Beck R de Craen AJ Slagboom EP Westendorp RG Pawelec G Infection with cytomegalovirus but not herpes simplex virus induces the accumulation of late-differentiated CD4+ and CD8+ T-cells in humans J Gen Virol 2011 92 Pt 12 2746 2756 21813708 10.1099/vir.0.036004-0

[B51] Appay V Dunbar PR Callan M Klenerman P Gillespie GM Papagno L Ogg GS King A Lechner F Spina CA Memory CD8+ T cells vary in differentiation phenotype in different persistent virus infections Nat Med 2002 8 4 379 385 10.1038/nm0402-379 11927944

[B52] Gamadia LE Rentenaar RJ Baars PA Remmerswaal EB Surachno S Weel JF Toebes M Schumacher TN ten Berge IJ van Lier RA Differentiation of cytomegalovirus-specific CD8(+) T cells in healthy and immunosuppressed virus carriers Blood 2001 98 3 754 761 10.1182/blood.V98.3.754 11468176

[B53] Pita-Lopez ML Gayoso I DelaRosa O Casado JG Alonso C Munoz-Gomariz E Tarazona R Solana R Effect of ageing on CMV-specific CD8 T cells from CMV seropositive healthy donors Immun Ageing 2009 6 11 10.1186/1742-4933-6-11 19715573 PMC2741428

[B54] Pourgheysari B Khan N Best D Bruton R Nayak L Moss PA The cytomegalovirus-specific CD4+ T-cell response expands with age and markedly alters the CD4+ T-cell repertoire J Virol 2007 81 14 7759 7765 10.1128/JVI.01262-06 17409149 PMC1933343

[B55] Fletcher JM Vukmanovic-Stejic M Dunne PJ Birch KE Cook JE Jackson SE Salmon M Rustin MH Akbar AN Cytomegalovirus-specific CD4+ T cells in healthy carriers are continuously driven to replicative exhaustion J Immunol 2005 175 12 8218 8225 16339561 10.4049/jimmunol.175.12.8218

[B56] Correa CB Kouri V Verdasquera D Martinez PA Alvarez A Aleman Y Perez L Viera J Gonzalez R Perez E HCMV seroprevalence and associated risk factors in pregnant women, Havana City, 2007 to 2008 Prenat Diagn 2010 30 9 888 892 10.1002/pd.2587 20715119

[B57] Douek DC McFarland RD Keiser PH Gage EA Massey JM Haynes BF Polis MA Haase AT Feinberg MB Sullivan JL Changes in thymic function with age and during the treatment of HIV infection Nature 1998 396 6712 690 695 10.1038/25374 9872319

[B58] Aspinall R Pido J Andrew D A simple method for the measurement of sjTREC levels in blood Mech Ageing Dev 2000 121 1–3 59 67 11164460 10.1016/s0047-6374(00)00197-4

[B59] Whitacre CC Reingold SC O’Looney PA A gender gap in autoimmunity Science 1999 283 5406 1277 1278 10.1126/science.283.5406.1277 10084932

[B60] Lio D Scola L Crivello A Colonna-Romano G Candore G Bonafe M Cavallone L Franceschi C Caruso C Gender-specific association between -1082 IL-10 promoter polymorphism and longevity Genes Immun 2002 3 1 30 33 10.1038/sj.gene.6363827 11857058

[B61] Balistreri CR Candore G Accardi G Bova M Buffa S Bulati M Forte GI Listi F Martorana A Palmeri M Genetics of longevity. data from the studies on Sicilian centenarians Immun Ageing 2012 9 1 8 10.1186/1742-4933-9-8 22524430 PMC3402998

[B62] Garcia B Neninger E de la Torre A Leonard I Martinez R Viada C Gonzalez G Mazorra Z Lage A Crombet T Effective inhibition of the epidermal growth factor/epidermal growth factor receptor binding by anti-epidermal growth factor antibodies is related to better survival in advanced non-small-cell lung cancer patients treated with the epidermal growth factor cancer vaccine Clin Cancer Res 2008 14 3 840 846 10.1158/1078-0432.CCR-07-1050 18245547

[B63] Candore G Balistreri CR Listi F Grimaldi MP Vasto S Colonna-Romano G Franceschi C Lio D Caselli G Caruso C Immunogenetics, gender, and longevity Ann N Y Acad Sci 2006 1089 516 537 10.1196/annals.1386.051 17261795

[B64] Vasto S Candore G Balistreri CR Caruso M Colonna-Romano G Grimaldi MP Listi F Nuzzo D Lio D Caruso C Inflammatory networks in ageing, age-related diseases and longevity Mech Ageing Dev 2007 128 1 83 91 10.1016/j.mad.2006.11.015 17118425

